# Knowledge and Attitude About Sexually Transmitted Infections Amongst Truck Drivers in Southern Punjab, Pakistan

**DOI:** 10.7759/cureus.1118

**Published:** 2017-03-26

**Authors:** Rizwan Ishtiaq, Ammar Asif, Abdur Rehman Jamil, Areba Irfan, Daniyal Ishtiaq

**Affiliations:** 1 General Medicine, Quaid-e-Azam Medical College, Bahawalpur, Pakistan; 2 General Medicine, Services Institute of Medical Sciences, Lahore, Pakistan; 3 General Medicine, Rawalpindi Medical College, Rawalpindi, Pakistan

**Keywords:** knowledge, attitude, sexually transmitted disease, hiv, condoms, truck drivers

## Abstract

**Introduction:**

There is very limited knowledge about sexually transmitted infections (STIs) transmission and prevention present amongst high-risk groups such as truck drivers in Pakistan because of lack of awareness and understanding about barrier techniques.

**Objective:**

The aim of this study was to collect and access the data gathered from truck drivers about symptoms of STIs, their attitude towards hazards of multiple sexual partners, homosexuality, transmission and consequences of STIs, and their perception about preventing it using condoms and other barrier methods.

**Methods:**

This study was conducted at small roadside tea stalls and local rest areas on Karachi road, Lodhran near the city of Bahawalpur in Southern Punjab, Pakistan. A structured questionnaire was designed, and 50 willing truck drivers of the city of Bahawalpur were included and interviewed. It was a cross-sectional study. Data was collected on knowledge about the STIs and use of barrier methods like condoms. Quantitative data was assessed and analyzed using SPSS version 23.0 (IBM, NY, USA).

**Results:**

Fifty truck drivers of Bahawalpur were interviewed via standardized questionnaire in this study. All of them provided answers about their knowledge of STIs. Twenty drivers (40%) reported burning micturition, and only two (four percent) knew the real cause of it. Thirty-two (64%) of them were well aware of the use of condoms. Thirty-eight (76%) truck drivers had the knowledge about the adverse effects of multiple sex partners.

**Conclusion:**

The truck drivers of Bahawalpur city are quite vulnerable to STIs and this demonstrates the importance of prevention programs that can target this particular group. A significant number of the respondents had serious gaps in their knowledge about STIs like acquired immunodeficiency syndrome (AIDS), especially its modes of transmission, signs, and symptoms. The knowledge of other routes of human immunodeficiency virus (HIV) transmission like needle sharing and blood transfusion, and precautionary steps should be given due respect in HIV/AIDS awareness programs.

## Introduction

Mobility is observed as a major factor for the spread of sexually transmitted infections (STIs) like human immunodeficiency virus (HIV) across different geographical locations [1]. Prevalence of diseases like HIV is more common in people living in rural settings, especially areas which are populated along the roads, because of low income, lack of education, awareness about barrier contraception and use of condoms, and increased amount of internally displaced persons due to natural calamities than when compared to urban areas [2]. Three hundred and forty million cases of STIs have been reported worldwide approximately in males and females in the age range of 15 to 49 years [3]. A sexually transmitted infection may be benign, or it can become chronic with long-term consequences including infertility, cervical cancers, or pelvic inflammatory disease [4].

Truck drivers are a group of people recognized to be active sexually; their long driving hours and exhausting working environment causing lethargy and mental fatigue incline them towards being sexually active [1]. This behavior and attitude pose them at high risk of acquiring STIs. In a recent study conducted in India, 30% of drivers and 50% of helpers reported unsafe sexual practices with sex workers exposing them to STIs [5].

The central concept in the basis for understanding the transmission dynamics of STI and HIV relates to the importance of a subset of the population (“critical community”) who have higher rates of partner change and concurrent sexual relationships with lack of awareness, little knowledge, and the need to travel long distances and overnight stay. Therefore, we selected truck drivers as they can be a source of transmission of STIs acting as national carriers. The aim of this study was to collect and access the data gathered from truck drivers about symptoms of STIs, their attitude towards hazards of multiple sexual partners, homosexuality, and their perception about preventing STIs using condoms and other barrier methods.

## Materials and methods

A structured questionnaire about the knowledge and symptoms of STIs was designed, and 50 willing truck drivers of the city of Bahawalpur were included and interviewed. The survey took place at seven tea stalls and local restaurants along the N-5 highway. These spots were chosen because of highly populated local villages and towns near them and low literacy rate. The study was carried out after getting approval from the institutional review board of Quaid-e-Azam Medical College, Bahawalpur. Data was collected from January 13, 2017, to January 19, 2017. Informed consent was taken from all truck drivers. A pre-designed custom-made questionnaire was used for data collection. It was a cross-sectional study. Qualitative data was collected on knowledge about the STI, its symptoms, and use of barrier methods like condoms. Quantitative data was assessed and analyzed using SPSS version 23.0 (IBM, NY, USA).

## Results

All of the truck drivers responded to our questions per their knowledge about STIs. The respondents did not find the practices like unprotected sexual intercourse, needle sharing while injecting drugs, and blood transfusion as risk factors, and hence the overall insight regarding the impact of HIV/AIDS was lacking. Detailed results are reported in Table 1. When asked about symptoms of urinary tract infections, 20 (40%) truck drivers had a history of burning micturition. When enquired about the cause of this complaint, only two (four percent) drivers knew the real cause of the burning micturition. Thirty-eight (76%) truck drivers knew the consequence of engaging in sexual activities with multiple partners. Thirty-two (64%) truck drivers in our study had some insight into the significance of condom use, and 15 (30%) reported using a condom before sexual intercourse. Fifteen (30%) truck drivers admitted engaging in anal sex with sex workers and out of these 15 drivers, six (12%) drivers used condoms. Eighteen drivers (36%) revealed engaging in homosexuality and out of these 18 truck drivers, eight (16%) drivers disclosed condom use.

**Table 1:** Attitude and behavior of truck drivers of Southern Punjab, Pakistan

Question	Frequency	Cumulative Percent
History of Burning Micturition	20	40%
History of Multiple Sexual Partners	38	76%
Knowledge about Condom and its Use	32	64%
Condom before Sexual Intercourse	15	30%
History of Anal Sex	15	30%
Condom use in Anal Sex	6	12%
History of Homosexual Activity	18	36%
Condom use in Homosexual Activity	8	16%

Twenty-eight (56%) drivers said that they have been driving for more than 20 years. The average income of the truck drivers was Rs. 17,000 (US$170) monthly. The data collected clearly shows that the truck drivers of Bahawalpur city are quite vulnerable to STI and demonstrate the importance of prevention programs that can target this particular group.

## Discussion

In Pakistan, apart from HIV/AIDS, the relevance of STIs in truck drivers has not been surveyed at any level. The necessary information regarding STIs also remains in the dark. The significant population share affected by HIV/AIDS is found in developing countries, which is further aggravated by the lack of health facilities, overcrowding, illiteracy, poverty, and underdevelopment.

The descriptive analysis gathered from the 50 truck drivers belonging to the region of Bahawalpur suggests that the awareness regarding HIV/AIDS is decumbent. The majority of the respondents had limited knowledge regarding the causes of HIV/AIDS, its presentation, mode of transmission, and who falls in the high-risk category. The respondents did not find the practices like unprotected sexual intercourse, needle sharing while injecting drugs, and blood transfusion as risk factors, and hence the overall insight regarding the impact of HIV/AIDS was lacking. Most of the interviewees only had a primary level of education, which contributes to the lack of awareness. Seventy percent population of Pakistan is rural. Twenty-two percent of the population is active economically, and more than 20% are unemployed. Almost 30% population of Pakistan earns less than US$1 [4]. The average income of the truck drivers was Rs. 17,000 (US$170) which meant they had limited access to the clinics dealing with the infectious diseases.

In support of this, a study was conducted in Togo, along the two main highways, in which the consistent use of condoms during intercourse was analyzed. It turns out that 34.8% of the driver population reported the steady use of condoms during sexual encounters in the last three months [1]. Although the numbers are small, but when a similar group was analyzed in 2008, the numbers came out to be 30.1% [6]. The low-set of condom use could explain why the incidence of HIV/AIDS is higher in this group of people. When dissecting the data in a little more depth, the drivers with the higher level of education reported the more persistent use of condoms in the last three months. Higher education makes it possible to understand the message given out by the health and education departments. Drivers with higher learning had a better understanding regarding the modes of transmission and how condoms play a significant role in limiting the spread of the disease. These groups of drivers were more aware of the importance of condom usage and reported persistent usage of condoms during intercourse [1]. Studies conducted in Kenya and Uganda reported a satisfying rate of 70% condom use among the truck drivers. Once again a higher level of education was identified as a major component of achieving a higher rate of condom use [7]. Another study conducted in Tanzania showed a direct correlation between education and higher usage of condoms [8]. A randomized controlled study carried out in Hong Kong depicted that the use of voluntary counseling and testing among the high-risk truck drivers had a significant impact on reducing the incidence of dangerous sexual practices and lessening the risk of HIV/AIDS [9]. Studies conducted along the Mexican borders reported similar results [10].

One study conducted by Raza, et al. assessed the knowledge, attitude, and practices towards HIV/AIDS in a group of 1088 respondents. He noticed that out of 733 males and 355 females, 95.2% men and 76.9% women were aware of the existence of HIV/AIDS. However, only 25.7% males and 21.4% females had the awareness regarding the cause of AIDS. Another aspect of the study was that the majority of the group population had an annulling attitude towards the patients with HIV/AIDS and felt that they should be isolated [11].

In south-eastern Tanzania, a study in which truck drivers, men, and women from rural areas were asked about heterosexual anal sex. The majority understood the concept and were aware of these practices. A common misconception was identified as this population group believed that STIs like AIDS reside only in wet areas like the vagina and having anal intercourse does not put them at risk. It was believed that anal intercourse is safer than vaginal sex and hence condoms were not needed. This belief was in contradiction to the medical point of view that anal intercourse is one of the risk factors for transmission of HIV. As a result of this study, it was found that heterosexual anal intercourse is practiced more often than anticipated. Study populations like these need to be counseled and educated regarding the risk factors of STIs. The false perception which exists in these study populations must be tackled properly [12]. A similar study conducted in India showed the same outcome. Another interesting aspect was that the information given regarding the transmission of STIs including HIV was more focused on vaginal intercourse. Practices like anal intercourse were not addressed. The efforts to increase the awareness via media and healthcare services regarding the risk factors for STIs need to include all types of sexual practices [13].

In the Unites States of America during the year 1995, information was collected from almost 71% of the population regarding the knowledge of HIV/AIDS. When the obtained information was analyzed, National Health Information System (NHIS) came with the hypothesis that the best outcome of information regarding the disease was the socio-economic status rather than the race, ethnicity, and exposure to AIDS [14]. In a similar study conducted in India, three out of 302 drivers were infected with AIDS due to the reasons aforementioned [15].

In our study, 50% of the participants responded that they screened blood for infection which could also limit the spread of the disease. Almost all had the information that homosexuality is one of the causes and they recognized this practice. Studies, which were done in Bangladesh and India, also showed that those truck drivers who were made aware regarding the preventive steps to take for HIV had a higher reported number for persistent usage of condoms. They also demonstrated the impact of the number of years being in the profession. Higher numbers (>15 years) are a protective factor for acquiring STIs [16-17]. Despite adequate knowledge regarding the use of condoms as a protective factor for HIV/AIDS, most of the truck drivers and female sex workers continue to engage in sexual activities without using condoms. It was found out that the majority of the study group reported heavy use of alcohol before indulging in sexual activity, which hampered their judgment. It was also said that condoms kill the sex mood, which leads to the drivers avoiding condom usage. Female sex workers would engage in sexual activity without using condoms to make extra money and to avoid being abused by their sexual partner. Special attention should be drawn to hurdles that stop sex workers using methods of protection so that the usage of protective methods can be increased by removing those hurdles [18-19]. A study exhibited the effectiveness of free counseling and setting up incognito testing for the drivers in decreasing the risk of sexual behavior. It henceforth mandates the need to set up counseling for the drivers and testing to check their HIV status [9,20].

## Conclusions

Awareness regarding STIs in Southern Punjab of Pakistan is substandard. A significant number of interviewees had a genuine gap in their understanding about signs and symptoms of HIV/STIs. They lack awareness about STI transmission and identification of risk factors. Counseling regarding the sexual practices needs to be addressed as well. Common misconceptions linked to the fact that HIV/STI can only be transmitted via vaginal intercourse need to be addressed. Almost all of them were aware of homosexuality and the adverse effects of multiple sex partners. Limitations in our study included smaller sample size and possibility of interviewer bias. The study gives us an idea of prospective studies assessing the knowledge and attitude of truck drivers or other populations with similar demographic characteristics.

It is the need of the hour to establish infectious disease clinics at basic health units to address the screening, treatment, and health education regarding STIs. Effective control measures should be instituted by creating awareness in the truck driver community about safe sex practices to prevent the establishment of high-risk behaviors. The knowledge of other routes of HIV transmission like needle sharing and blood transfusion should be given due importance in HIV/AIDS awareness programs. Truck drivers should be educated regarding the identification of risk factors and habits of safe sexual practices. Infected patients should be counseled about regular medical checkups at health centers until the disease is resolved. Regular screening should be included in the clinical practice to limit the spread of such infections. A National Health Education campaign should be initiated to promote condom use. Individuals affected with STIs should be notified to the public health authority.
